# Fluorine Mass Balance, including Total Fluorine, Extractable Organic Fluorine, Oxidizable Precursors, and Target Per- and Polyfluoroalkyl Substances, in Pooled Human Serum from the Tromsø Population in 1986, 2007, and 2015

**DOI:** 10.1021/acs.est.3c03655

**Published:** 2023-09-25

**Authors:** Lara Cioni, Merle Plassmann, Jonathan P. Benskin, Ana Carolina M. F. Coêlho, Therese H. Nøst, Charlotta Rylander, Vladimir Nikiforov, Torkjel M. Sandanger, Dorte Herzke

**Affiliations:** † NILU, Fram Centre, Tromsø NO-9296, Norway; ‡ Department of Community Medicine, UiT − The Arctic University of Norway, Tromsø NO-9037, Norway; § Department of Environmental Science, Stockholm University, Stockholm SE-106 91, Sweden; ∥ Norwegian Institute for public Health, Oslo NO-0213, Norway

**Keywords:** human exposure, PFAS, PFAA precursors, TF, EOF, TOP assay, time trend

## Abstract

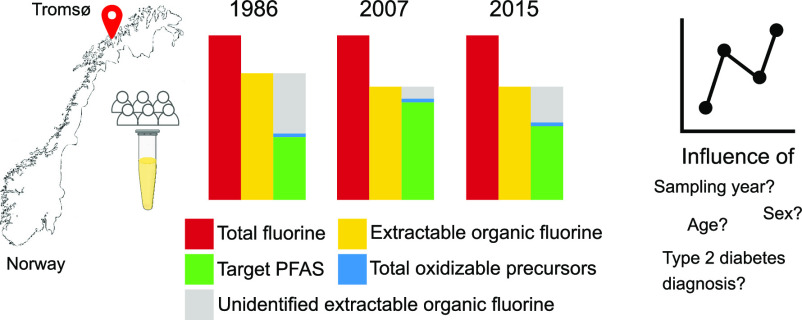

Of the thousands of per- and polyfluoroalkyl substances (PFAS) known to exist, only a small fraction (≤1%) are commonly monitored in humans. This discrepancy has led to concerns that human exposure may be underestimated. Here, we address this problem by applying a comprehensive fluorine mass balance (FMB) approach, including total fluorine (TF), extractable organic fluorine (EOF), total oxidizable precursors (TOP), and selected target PFAS, to human serum samples collected over a period of 28 years (1986, 2007, and 2015) in Tromsø, Norway. While concentrations of TF did not change between sampling years, EOF was significantly higher in 1986 compared to 2007 and 2015. The ∑_12_PFAS concentrations were highest in 2007 compared to 1986 and 2015, and unidentified EOF (UEOF) decreased from 1986 (46%) to 2007 (10%) and then increased in 2015 (37%). While TF and EOF were not influenced by sex, women had higher UEOF compared to men, opposite to target PFAS. This is the first FMB in human serum to include TOP, and it suggests that precursors with >4 perfluorinated carbon atoms make a minor contribution to EOF (0–4%). Additional tools are therefore needed to identify substances contributing to the UEOF in human serum.

## Abstract 2

The combined application of targeted and group-wise analyses on pooled serum samples enables the evaluation of the contribution of known and so far unidentified fluorinated compounds in human serum through time.

## 1 Introduction

Per- and polyfluoroalkyl substances (PFAS) are a group of synthetic chemicals with over 200 applications in industrial processes and consumer products.^1^ Due to their widespread use and high persistence, PFAS have been observed throughout the environment, including wildlife and human blood globally.^2^ PFAS ubiquity has led to concerns surrounding their ongoing production and use, in particular because some of them have been linked to adverse health effects, both in epidemiological and animal studies.^3^ These effects include impaired immune system, thyroid dysfunction, liver disease, lipid dysregulation, kidney disease, and adverse reproductive and developmental outcomes.^3^

PFAS production and use restrictions were introduced in the United States and European Union in early 2000s, following the phase-out of perfluorooctanesulfonic acid (PFOS) and perfluorooctanoic acid (PFOA) by 3M.^4,5^ PFOS was subsequently added to the Stockholm Convention on Persistent Organic Pollutants (POPs) in 2009 followed by PFOA in 2019.^6,7^ While PFOA and PFOS concentrations in human blood have declined globally in response to these initiatives,^2,5^ longer perfluoroalkyl carboxylic acids (PFCA) are not following the same trend.^5,8^ Moreover, as fluorochemical manufacturers shift toward production of unregulated PFAS, novel PFAS may become increasingly relevant for human exposure.^9^

Of the ∼4600 PFAS registered on the global market in 2018,^10^ ≤1% are routinely analyzed in human biomonitoring studies.^2,11^ This discrepancy has led to doubts about whether targeted methodologies are sufficient to describe the full extent of PFAS exposure. Indeed, a growing number of fluorine mass balance (FMB) studies in human blood have quantified large fractions of extractable organic fluorine (EOF) that cannot be explained by targeted PFAS analyses.^12−18^ One possible explanation for this gap are perfluoroalkyl acids (PFAA) precursors, such as perfluorooctane sulfonamides, fluorotelomer alcohols, and polyfluoroalkyl phosphate esters. Many of these substances have been detected in human blood using targeted methodologies,^19^ but as-of-yet unidentified precursors may also be important. The total oxidizable precursor (TOP) assay, in which PFAA concentrations are measured before and after controlled oxidation,^20^ offers a promising means for quantifying the total contribution from both known and unknown precursors. While the TOP assay has been used successfully to determine PFAA precursors in environmental samples^21−26^ and consumer products,^27−31^ there are few examples of its application to human serum,^32,33^ and in particular no examples when used in conjunction with an FMB.

Here, we build upon previous analyses of PFAS time-trends in serum from the Tromsø Study, which showed that PFAS concentrations in the Tromsø population changed according to the history of production and use of these chemicals and that time-trends differed depending on birth cohort, age group, and study design.^8,34^ In the present study, serum samples from the Tromsø Study collected in 1986, 2007, and 2015 were pooled and for the first time analyzed for total fluorine (TF), EOF, TOP, and selected target PFAS. Through the combined application of a set of targeted and group-wise analyses, we aimed to evaluate exposure to total fluorine and known and unknown organic fluorinated compounds over time with respect to sex and age.

## 2 Materials and Methods

Information on chemicals and consumables is provided in the SI.

### 2.1 Serum Samples and Pooling Strategy

The Tromsø Study is a cohort study of the population of Tromsø, the largest city in Northern Norway. Details on the Tromsø Study are provided by Jacobsen et al.^35^ The study obtained informed consent from all participants and was approved by the Regional Committee for Medical Research Ethics (REK, case number: 2020/13188).

The present work utilized 529 individual Tromsø Study serum samples collected in 1986 (*n* = 201), 2007 (*n* = 198), and 2015 (*n* = 130) (Figure S1). The samples were selected based on a case/control study design on type-2 diabetes: the cases (1986 [*n* = 84], 2007 [*n* = 102], 2015 [*n* = 62]) were diagnosed between 2001 and 2007, while the controls (1986 [*n* = 117], 2007 [*n* = 97], 2015 [*n* = 68]) had no diagnosis recorded in the local diabetes registry. The selected samples included 104 women and 97 men in 1986, 113 women and 86 men in 2007, and 72 women and 58 men in 2015. The age of the individuals ranged from 17 to 61 years old in 1986 (mean: 46), from 38 to 81 in 2007 (mean: 67), and from 46 to 89 in 2015 (mean: 72).

**Figure 1:**
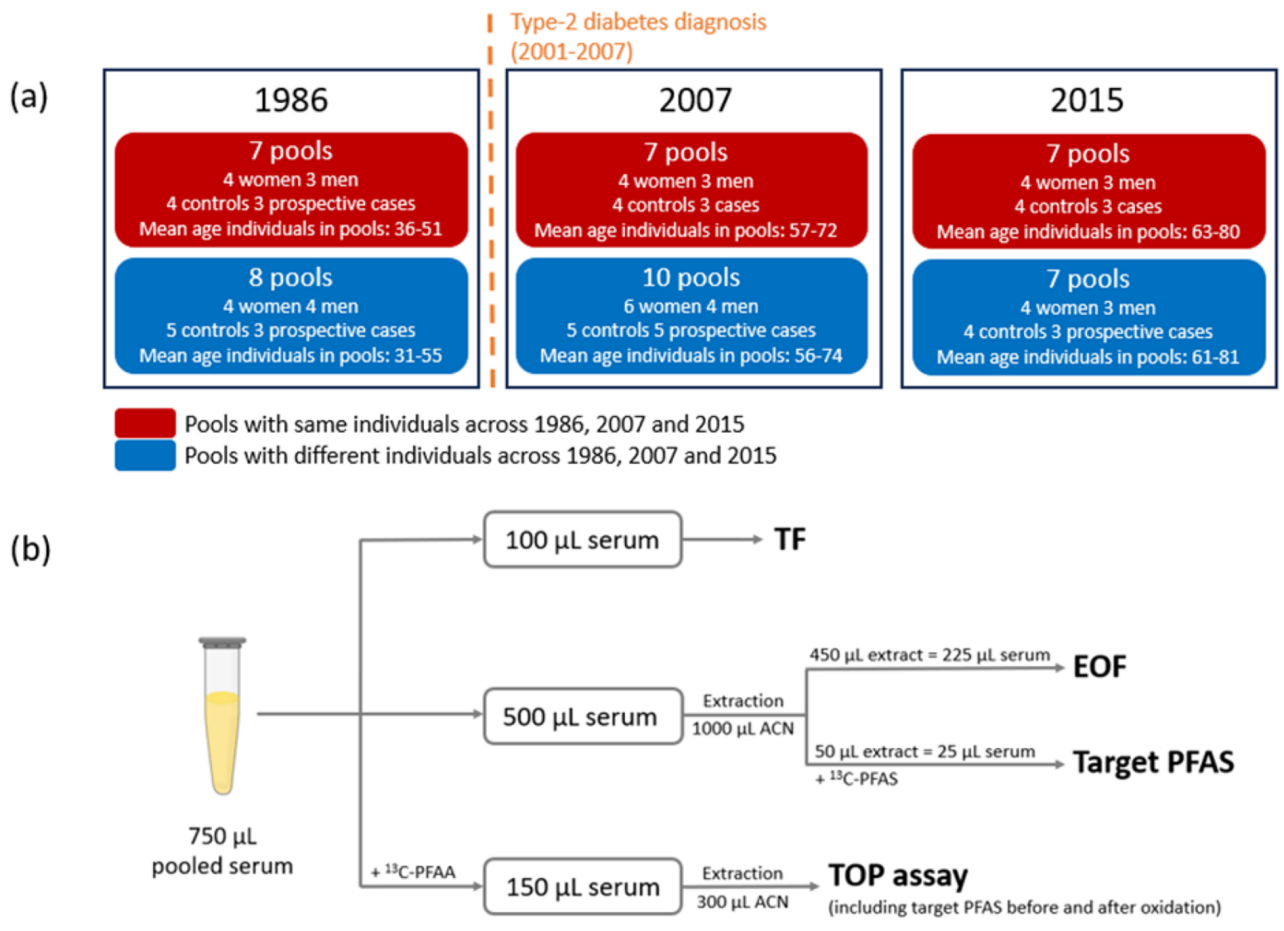
(a) Pooling strategy summary and (b) fluorine mass balance approach.

From this selection, 472 individual samples (1986 [*n* = 167], 2007 [*n* = 175], 2015 [*n*= 130]) were pooled based on sampling year, sex, age, and type-2 diabetes diagnosis (Figure 1, Table S1). Sampling year, sex, and age were chosen as variables for pooling because these are known to influence PFAS concentrations in human blood. Type-2 diabetes diagnosis was used as a variable for pooling because some studies have reported associations between this end point and PFAS concentrations, but it is important to note that evidence for these associations is contradictory.^36^ Pools 1 to 7 at each sampling year included the same individuals in 1986, 2007, and 2015. To have the largest possible number of pools including the same individuals, these pools were obtained mixing variable volumes (50, 100, or 150 μL) of individual serum samples but keeping the volume per individual constant throughout the sampling years. For the remaining pools, it was not possible to follow the same individuals through time and 15 participants (with matching sampling year, sex, age, and type-2 diabetes diagnosis) were included in each pool mixing 50 μL of serum per individual.

### 2.2 Fluorine Mass Balance

Each pool was analyzed using a combination of analytical techniques to evaluate different fluorine fractions (Figure 1). The pools were split into three portions: (1) 100 μL for TF, (2) 500 μL for EOF, and (3) 150 μL for the TOP assay. Target PFAS analysis was performed on the TOP assay extracts (before and after oxidation) and on the EOF extracts after addition of internal standard.

#### 2.2.1 Total Fluorine

For TF measurements, 100 μL of serum was transferred to a sampling boat for analysis using a Thermo-Mitsubishi combustion ion chromatograph (CIC) with the method described by Miaz et al.,^15^ which was previously demonstrated to produce fluorine-specific responses.^37^ Details about quality control measures (including calibration, blank values, LODs, accuracy, and precision evaluation) are reported in the SI.

#### 2.2.2 Extractable Organic Fluorine

For EOF determination, 500 μL of serum was transferred to Eppendorf tubes and extracted once with 1 mL of ACN. Samples were vortexed and sonicated (10 min) 3 times, and after centrifugation at 10,000 rpm for 10 min, supernatants were transferred to 2 mL glass vials. EOF analyses were performed on 450 μL of the extracts with the same CIC used for TF analyses and the method described by Miaz et al.^15^ Details about quality control measures (including calibration, blank values, LODs, evaluation of PFOS recovery, reproducibility, and removal of fluoride upon extraction) are reported in the SI.

#### 2.2.3 Total Oxidizable Precursor Assay

For the TOP assay, 150 μL of serum was processed using a previously published protocol.^32^ Briefly, samples were spiked with ^13^C-PFAA and extracted with ACN. After vortexing, sonication, and centrifugation, the supernatant was collected and split into two portions: one for target analyses before oxidation and one was oxidized for TOP determination. Prior to oxidation, ACN was removed by evaporation, and the dry extracts were reconstituted with 0.8 M Na_2_S_2_O_8_ and 10 M NaOH. Post oxidation, the samples were acidified and extracted with MTBE. Aliquots of the organic phase were transferred to vials with insert and spiked with recovery standard and 2% ammonia in methanol. The MTBE was evaporated prior analyses. Details about quality control measures (including blanks, LODs, and recoveries before and after the TOP assay, and summary of method validation with model precursors) are reported in the SI.

#### 2.2.4 Target PFAS

Target analyses on the EOF extracts included 54 PFAS (Table S5) and were performed using a Dionex UltiMate 3000 Ultrahigh performance liquid chromatograph coupled to a Q Exactive HF hybrid Quadrupole-Orbitrap mass spectrometer (Thermo Fisher Scientific, Waltham, MA, USA) as described elsewhere.^15^ For these analyses, 50 μL of EOF extracts was mixed with 10 μL of internal standard and 50 μL of 4 mM NH_4_OAc in Milli-Q water. Since the internal standard was added after extraction, these concentrations were not recovery corrected and were only used for FMB calculations. LODs and accuracy of these analysis are reported in the SI.

Target analyses on the TOP assay extracts included 34 PFAS and were performed using a quaternary Accela 1250 pump with a PAL Sample Manager coupled to a Vantage TSQ MS/MS (Thermo Fisher Scientific, Waltham, MA, USA) as described elsewhere.^32^

After oxidation, the extracts were also analyzed for C_2_ and C_3_-PFAA using a Raptor Polar X column. Details about these analyses and the quality control measures (including blank concentrations and LODs) are provided in the SI.

### 2.3 Data Treatment

#### 2.3.1 Fluorine Mass Balance Calculations

EOF values were subtracted from TF concentrations to estimate the amount of inorganic and nonextractable organic fluorine. For this comparison, samples with TF values below the LOD were excluded. To estimate the unidentified portion of EOF (UEOF), the ∑_12_PFAS concentrations obtained from the EOF extracts (Table S6) were subtracted from the EOF concentrations. For this comparison, PFAS concentrations were converted to fluorine equivalents using eq S1. PFAS concentrations below the LOD were set to LOD/√2. The ∑_12_PFAS concentrations and detection frequencies from the EOF extracts are lower and less accurate than those from the TOP assay extracts (Table 2 and S6) because of the lack of recovery correction for procedural losses. However, the use of ∑_12_PFAS concentrations not corrected for recovery for FMB calculations provides a more representative and accurate result in terms of mass balance. This is because the EOF concentrations cannot be recovery corrected since the addition of an internal standard before extraction would increase the LOD and it is not possible to correct for the recovery of unknown fluorinated chemicals present.

The total amount of oxidizable precursors (ΔPFAA) was estimated as described by Coêlho et al.^33^ To establish whether there was an increase in PFAA concentrations after oxidation, the ratio between the concentration after oxidation and the concentration before oxidation (PFAA_after-TOPA_/PFAA_before-TOPA_) was calculated. To avoid the possibility that apparent changes were influenced by analytical uncertainties, a cutoff of 20% change in PFAA concentrations was applied. Specifically, if the ratio was ≥1.2, the difference (ΔPFAA) was calculated as the PFAA concentration after oxidation minus the PFAA concentration before oxidation. If the ratio was <1.2, ΔPFAA was set to zero.

To estimate the contribution of TOP to EOF, ΔPFAA concentrations were converted to F equivalents with the same equation used for target PFAS (eq S1). This comparison has some uncertainty because the TOP assay data are corrected for procedural losses but the EOF data are not.

#### 2.3.2 Statistical Analyses

Statistical analyses were performed using R version 4.1.2 (R Core Team). Prior to statistics calculations, concentrations below the LOD were substituted with LOD/√2. Differences in TF, EOF, ∑_12_PFAS, unidentified EOF, and TOP between sampling years, sex, and age (as weighted mean of the age of the individuals in the pools expressed in years) groups were assessed by multiple linear regression using eq S2. When sex was a significant predictor, differences in concentrations between men and women at each sampling year were assessed adding an interaction term (eq S3). The inclusion of the type-2 diabetes diagnosis (case/control status) to the multiple linear regression model was tested by using Akaike information criterion (AIC) model selection. Since the model with the lowest AIC score never included the diabetes diagnosis variable, this was not included. TF, EOF, and ∑_12_PFAS concentrations were log-transformed before performing regression analyses. Statistical significance was set at *p* < 0.05. Posthoc power calculations were performed using the pwr package.

## 3 Results and Discussion

### 3.1 Total Fluorine

**Figure 2:**
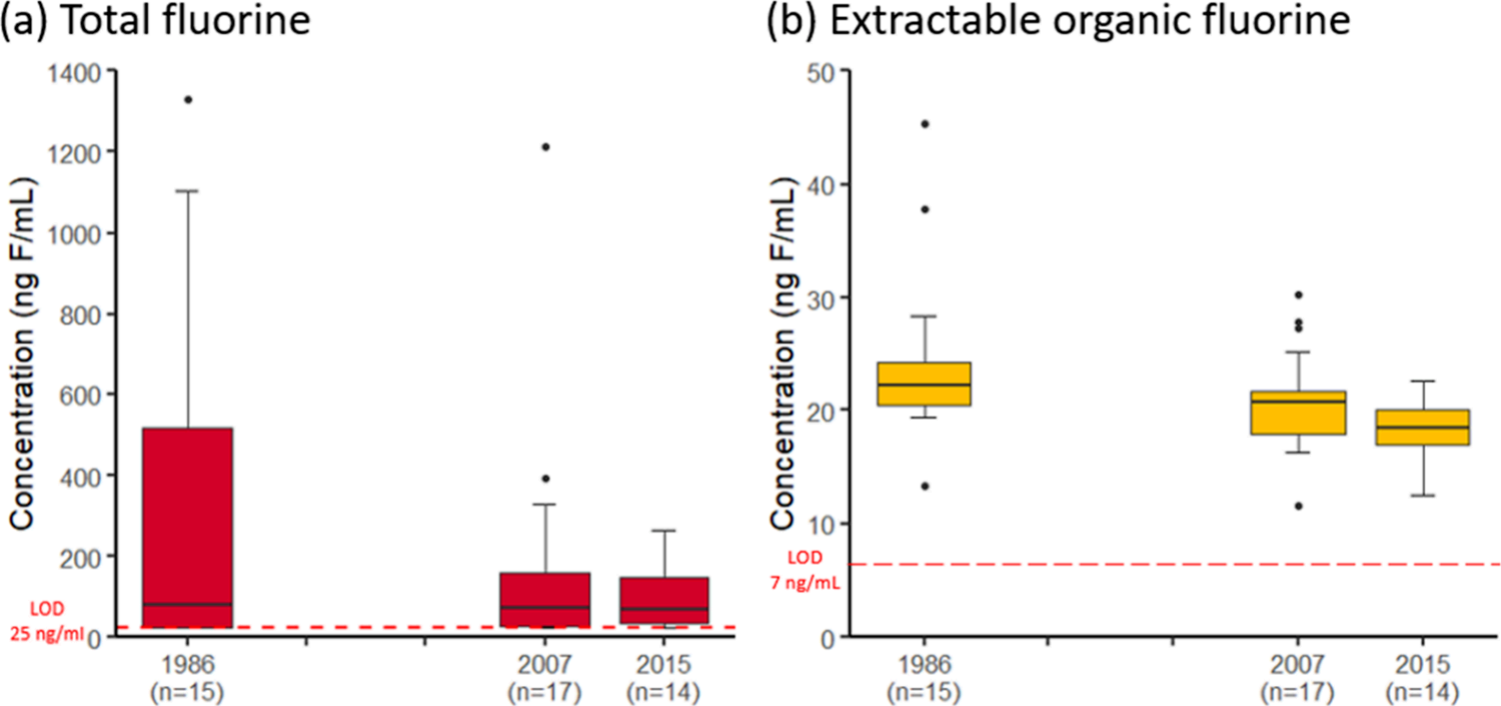
(a) TF and (b) EOF concentrations (ng F/mL) in pooled serum samples from the Tromsø Study collected in 1986, 2007, and 2015 (*n* = number of pools). The boxes represent the interquartile range between the 25th and 75th percentiles, containing the middle 50% data. The line in the boxes represents the median. The whiskers extend from the smallest observation greater than/equal to the 25th percentile minus 1.5 times the interquartile range to the largest observation lower than/equal to the 75th percentile plus 1.5 times the interquartile range. The points outside the whiskers represent outliers with values outside these limits.

TF in pooled serum from the Tromsø Study ranged from <25 to 1330 ng F/mL, with a narrower range observed in 2015 compared to 1986 and 2007 (Figure 2a, Table S7). The percentage of pools with TF below the LOD (25 ng F/mL) was 33% in 1986, 24% in 2007, and 7% in 2015. Based on multiple linear regression, there were no significant differences in TF concentrations between 1986, 2007, and 2015 and no significant effect of sex and age (Table S8). For TF, the time differences observed in the pools with the same individuals were not consistent, and this could be explained by TF being a sum parameter, including both inorganic and organic compounds containing fluorine for which the contribution might vary between individuals. In two of these pools, the concentration temporal changes clearly differed from the rest of the pools with the same individuals because these were below or close to the LOD at all sampling years (Figure S2).

In contrast with the results of our study, Miaz et al.^15^ observed declining TF concentrations in pooled serum samples from Swedish women collected between 1996 and 2017 (3.2% decline per year), although in that study the cohort was consuming PFAS-contaminated drinking water up until mid-2012.

The range of observed TF concentrations in 1986 and 2007 was wider than those reported in the literature, but in 2015, it was comparable (Table S7). However, the mean concentrations of TF in 1986, 2007, and 2015 were comparable to those reported for blood samples from China in 2008 and lower than those reported for serum from Japan in 2003–2004 and plasma from the USA in 2001 (Table S7).

### 3.2 Extractable Organic Fluorine

EOF in serum from the Tromsø Study ranged from 12.6 to 45.3 ng F/mL across all time-points (Figure 2b, Table S7). Unlike TF, EOF was detected in all pools (LOD = 7 ng F/mL). EOF concentrations in 1986 were significantly higher than those in 2007 and 2015, while no significant differences were found between 2007 and 2015 (Table S7, Table S8).

For EOF, the time differences observed in the pools from the same individuals were not consistent (Figure S2), and as for TF, this could be explained by EOF being a sum parameter, including potentially different PFAS and organofluorine chemicals.

The EOF concentrations observed in our study were in the same range as those observed in plasma from Germany collected between 1982 and 2009 and in pooled serum samples from Swedish women collected between 1996 and 2017. However, no significant time trends were observed for EOF in the German (1982–2009) and Swedish (1996–2017) samples.^14,15^ EOF concentrations in 2007 and 2015 were also comparable to those in whole blood collected in China in 2004 and in Sweden in 2015 and between 2018 and 2019. The EOF at all sampling years was higher than in whole blood from Japan (2003) and pooled serum from Austria (2021) but lower than the EOF in plasma from the USA (2001) and in whole blood from people living in Ronneby, where drinking water has been contaminated from firefighting foams (Table S7). However, apparent differences in EOF measurements between studies must be interpreted cautiously since different extraction methods may perform differently for individual fluorinated substances.^38^ In addition, different EOF values can be measured from different blood fractions since some PFAS, for example, perfluorohexanoic acid (PFHxA) and perfluorooctane sulfonamide (FOSA), bind minimally to serum proteins and are usually detected in whole blood rather than serum or plasma.^39^

Based on multiple linear regression, sex and age were not associated with EOF. This observation agrees with EOF measurements in samples from China that also showed no sex- and age-related differences.^13^ On the contrary, EOF concentrations in samples from Sweden in 2021 were higher in women compared to men.^16^

### 3.3 Total Oxidizable Precursors

The pooled samples from the Tromsø Study were also analyzed with the TOP assay to evaluate the contribution of oxidizable precursors. Even if the increases in PFAA concentrations (ΔPFAA) were low (0.02–1.85 ng/mL), all pools (except one from 2007) contained detectable oxidizable precursors (Table 1). No significant differences in TOP concentrations were found between 1986, 2007, and 2015 and sex and age did not influence the TOP measured (Table S8).

**Table 1:** Differences in PFAA Concentrations before and after TOP Assay Oxidation (ΔPFAA = PFAA_after-TOP_–PFAA_before-TOP_) in Pooled Serum Samples from the Tromsø Study (*n* = Number of Pools)^a^

	1986 (*n* = 15)				2007 (*n* = 17)				2015 (*n* = 14)			
	DF (%)	median	mean	range	DF (%)	median	mean	range	DF (%)	median	mean	range
ΔPFPeA	5/15 (33%)	0.00	0.03	0.00–0.09	1/17 (6%)	0.00	0.01	0.00–0.11	0/14 (0%)			
ΔPFHxA	0/15 (0%)				1/17 (6%)	0.00	0.08	0.00–1.32	0/14 (0%)			
ΔPFHpA	7/15 (47%)	0.00	0.04	0.00–0.12	0/17 (0%)				0/14 (0%)			
ΔPFOA	5/15 (33%)	0.00	0.23	0.00–1.00	0/17 (0%)				0/14 (0%)			
ΔPFNA	4/15 (27%)	0.00	0.04	0.00–0.18	1/17 (6%)	0.00	0.02	0.00–0.40	1/14 (7%)	0.00	0.02	0.00–0.36
ΔPFDA	1/15 (7%)	0.00	0.00	0.00–0.05	2/17 (12%)	0.00	0.01	0.00–0.15	0/14 (0%)			
ΔPFUnDA	3/15 (20%)	0.00	0.03	0.00–0.15	0/17 (0%)				2/14 (14%)	0.00	0.02	0.00–0.18
ΔPFDoDA	9/15 (60%)	0.04	0.03	0.00–0.09	12/17 (71%)	0.06	0.07	0.00–0.15	6/14 (43%)	0.00	0.03	0.00–0.14
ΔPFBS	0/15 (0%)				0/17 (0%)				13/14 (93%)	0.21	0.19	0.00–0.35
ΔPFHxS	4/15 (27%)	0.00	0.04	0.00–0.26	0/17 (0%)				0/14 (0%)			
ΔPFHpS	9/15 (60%)	0.05	0.08	0.00–0.21	14/17 (82%)	0.18	0.19	0.00–0.43	8/14 (57%)	0.11	0.11	0.00–0.32
ΔPFAA	15/15	0.43	0.52	0.17–1.16	16/17	0.26	0.38	0.00–1.85	14/14	0.36	0.38	0.13–0.66

a DF = detection frequency: number and % of pools with an increase in concentration after oxidation (PFAS_after-TOP_/PFAS_before-TOP_ ≥ 1.2)

For TOP, the time differences observed in pools from the same individuals were not consistent, and this could be due to a higher variability in precursors exposure or to the low concentrations of precursors present. Additionally, for this method a higher variability compared to target PFAS measurements is expected since the TOP concentrations are estimated by comparing PFAA concentrations before and after oxidation (Figure S2).

Increases in concentrations after oxidation were observed for eight PFCA and three PFSA (Table 1). Perfluorododecanoic acid (PFDoDA), perfluorobutanoic sulfonic acid (PFBS), and perfluoroheptanoic sulfonic acid (PFHpS) were the only compounds to display increased concentrations after oxidation in more than 50% of the pools at least one time-point. While ΔPFDoDA and ΔPFHpS were observed at all the examined time-points, ΔPFBS was detected in only serum pools from 2015. Increases in concentrations after oxidation were also detected for perfluoropentanoic acid (PFPeA), PFHxA, perfluoroheptanoic acid (PFHpA), PFOA, perfluorononanoic acid (PFNA), perfluorodecanoic acid (PFDA), perfluoroundecanoic acid (PFUnDA), and perfluorohexanoic sulfonic acid (PFHxS) but in a limited number of pools. Increases in concentrations of multiple PFAA following oxidation were more common than increases in only one PFAA, even if eight pools showed an increase only in PFHpS (five samples), PFDoDA (two samples), and PFBS (one sample) (Figure S3). The pattern of oxidation products differed from those observed for model precursors spiked into human serum^32^ and could not be used to tentatively identify precursors in serum from the Tromsø Study. However, even if the structure of the precursor(s) is lost upon oxidation, the profile of the oxidation products offers clues about the chain length of the precursor and the presence of sulfonic groups. For example, ΔPFDoDA points to the presence of precursors with 11 or more perfluorinated carbons, while ΔPFBS and ΔPFHpS suggest the presence of precursors containing sulfonic groups attached to four or seven perfluorinated carbons.^32^

The TOP assay has previously been applied to plasma samples collected between 2003 and 2006 from Norwegian women.^33^ The patterns of PFAA that increased after oxidation were different from those observed in this study. In contrast to our study, no increases in the levels of PFDoDA and PFBS were observed. Also, in the Tromsø Study pools, the concentrations of branched isomers of PFOA and PFOS did not increase after the TOP assay and the detection of ΔPFHpA, ΔPFNA, ΔPFDA, and ΔPFUnDA was limited, while in the plasma collected from Norwegian women, seven PFAA increased after oxidation (PFHpA, branched-PFOA, PFNA, PFDA, PFUnDA, PFHpS, and branched PFOS) with the greatest concentration differences observed for PFHpA, branched PFOA, and PFDA. There are several possible explanations for these differences. First, there could be differences in the exposure among the studied groups. The samples in this study were collected from both men and women living in Tromsø, while in the Coêlho et al. study, samples were collected only from women but from all over Norway. Additionally, the sampling years were different in the two studies. Second, the use of serum in the present study and plasma in the other could lead to the detection of different precursors. Another possible explanation could be the different extraction methods used in the two studies, resulting in different extraction effectiveness of the precursors present.

### 3.4 Target PFAS

In the pooled samples, 12 out of 54 target PFAS were detected: six PFCA (PFHpA, PFOA, PFNA, PFDA, PFUnDA, and PFDoDA), three PFSA (PFHxS, PFHpS, and PFOS), and three sulfonamidoacetic acids (FOSAA, Me-FOSAA, and Et-FOSAA). Branched isomers were above the LOD only for PFOS. It is interesting to note that, in agreement with the TOP assay results showing low concentrations of precursors, no precursors included in the target analyses other than the sulfonamidoacetic acids (including fluorotelomer sulfonates, fluorotelomer carboxylic acids, and fluorotelomer phosphate esters) were detected in pooled serum. Other biomonitoring studies investigating the presence of these precursors in human blood have also reported no detection or detection at trace levels (pg/mL).^40−42^ However, some of these precursors have been widely detected in consumer products, such as cosmetics and food packaging.^43,44^ This discrepancy between wide detection in consumer products and low detection in human blood might be due to a low uptake potential, rapid metabolism, or elimination of precursors in humans, but the contribution of precursor metabolism to indirect PFAA exposure remains unknown.^19,45,46^

Based on the analysis of the Tromsø Study pools, the ∑_12_PFAS concentrations in 2007 were significantly higher than those in 1986 and 2015 (Table 2, Table S8). Focusing on individual PFAS changes over time, concentrations of all PFAA in 2007 were higher than in 1986, except for PFHpA. Between 2007 and 2015, PFSA (PFHxS, PFHpS, and PFOS) and PFOA concentrations decreased, as opposed to the longer chained PFCA (PFNA, PFDA, PFUnDA, and PFDoDA), for which concentrations increased. Concentrations of the sulfonamidoacetic acids increased from 1986 to 2007, but none was detected in 2015. PFHpA concentrations were comparable in 1986, 2007, and 2015 (Table 2, Figure S4).

**Table 2:** Concentrations (ng/mL) in Pooled Serum Samples from the Tromsø Study before TOP Assay Oxidation (*n* = Number of Pools)^a^

	1986 (*n* = 15)				2007 (*n* = 17)				2015 (*n* = 14)			
	DF	median	mean	range	DF	median	mean	range	DF	median	mean	range
PFHpA	13/15	0.06	0.06	<0.02–0.25	17/17	0.06	0.05	0.03–0.08	13/14	0.05	0.04	<0.02–0.09
PFOA	15/15	2.44	2.35	1.53–3.30	17/17	3.59	3.66	3.26–4.55	14/14	2.34	2.46	1.86–3.34
PFNA	15/15	0.56	0.59	0.39–1.08	17/17	1.71	1.65	1.27–2.31	14/14	1.99	2.03	1.43–1.89
PFDA	15/15	0.20	0.19	0.11–0.37	17/17	0.65	0.64	0.33–1.09	14/14	0.75	0.76	0.41–1.32
PFUnDA	15/15	0.61	0.63	0.48–1.05	17/17	1.04	1.02	0.55–2.16	14/14	1.08	1.06	0.43–1.96
PFDoDA	5/15	<0.02	<0.02	<0.02–0.08	9/17	0.06	0.03	<0.02–0.14	11/14	0.06	0.05	<0.02–0.13
PFHxS	15/15	0.74	0.69	0.38–1.17	17/17	2.31	2.37	1.61–6.36	14/14	1.99	2.13	1.18–4.74
PFHpS	10/15	0.10	0.07	<0.03–0.32	17/17	0.29	0.29	0.10–0.61	14/14	0.23	0.24	0.10–0.58
br-PFOS	15/15	9.53	9.16	6.63–12.3	17/17	14.9	14.5	10.6–20.5	14/14	8.92	9.73	7.54–14.3
lin-PFOS	15/15	15.9	15.5	12.0–21.5	17/17	22.6	23.5	15.8–42.6	14/14	15.5	17.3	9.34–29.0
FOSAA	14/15	0.12	0.12	<0.04–0.32	0/17				0/14			
Me-FOSAA	15/15	0.20	0.18	0.07–0.35	17/17	0.11	0.11	0.05–0.21	0/14			
Et-FOSAA	15/15	0.43	0.41	0.25–0.58	0/17				0/14			
∑_12_PFAS	15/15	30.9	30.2	23.7–40.3	17/17	47.0	48.2	38.7–75.7	14/14	34.0	36.3	22.9–52.4

a DF = detection frequency: number of pools with PFAS concentration > LOD.

The increase in ∑_12_PFAS and individual PFAA concentrations between 1986 and 2007 points to increased exposure between these years. However, we know from previous PFAS analyses in serum from the Tromsø Study, including individual samples from 1994 and 2001, that target PFAS concentrations peaked in 2001 with an increase between 1979 and 2001 followed by a decrease between 2001 and 2007.^8,34^ Divergent trends between PFOA and longer chained PFCA were also reported in the aforementioned studies: while PFOA concentrations peaked in 2001, long chained PFCA were increasing between 2001 and 2007. These trends in the Tromsø Study samples have been shown to follow trends of PFAS production and use.^26^

The ∑_12_PFAS concentrations in the pools with the same individuals followed the temporal changes described by multiple linear regression, except in one pool, that showed comparable ∑_12_PFAS concentrations in 1986 and 2007. This deviation could be due to this pool containing a lower number of individuals (10) compared to the other ones (11–14). With a lower number of individuals in a pool, even just one outlier could have a larger impact on the measured target PFAS concentrations (Figure S2).

Mean age of the individuals in the pools was a predictor of the ∑_12_PFAS concentrations between 1986 and 2015 (Table S8). The highest ∑_12_PFAS concentrations were found in the pools with the highest mean age (Figure S5). This has been explained by higher exposure in the older birth cohorts compared to the younger ones due to the history of changing PFAS production.^8^

Men had significantly higher ∑_12_PFAS concentrations than women (Table S8). When looking at the difference in ∑_12_PFAS concentrations at each time-point, men had significantly higher concentrations only in 2007 (Table S9). However, the difference might not be observed at all time-points due to limited statistical power. To obtain a power of 80% with a large effect size (0.35), at least 39 samples are necessary, and the number of pools at each time-point is lower than this value. Higher concentrations in men compared to women were observed for most of the individual PFAS (PFOA, PFNA, PFDA, PFUnDA, PFDoDA, PFHxS, PFHpS, and PFOS), but comparable concentrations were observed for PFHpA and the three sulfonamido acetic acids (FOSAA, Me-FOSAA, and Et-FOSAA) (Figure S4). Higher PFAS concentrations in men compared to women were already reported in the Tromsø Study by Berg et al.,^34^ which also noted higher PFAS concentrations in women that had not given birth compared to multiparous women. Placental transfer,^47−52^ breast feeding,^53−56^ and menstruation^57−60^ are known PFAS elimination pathways in women and could all contribute to explain sex differences in PFAS concentrations.

### 3.5 Fluorine Mass Balance

**Figure 3:**
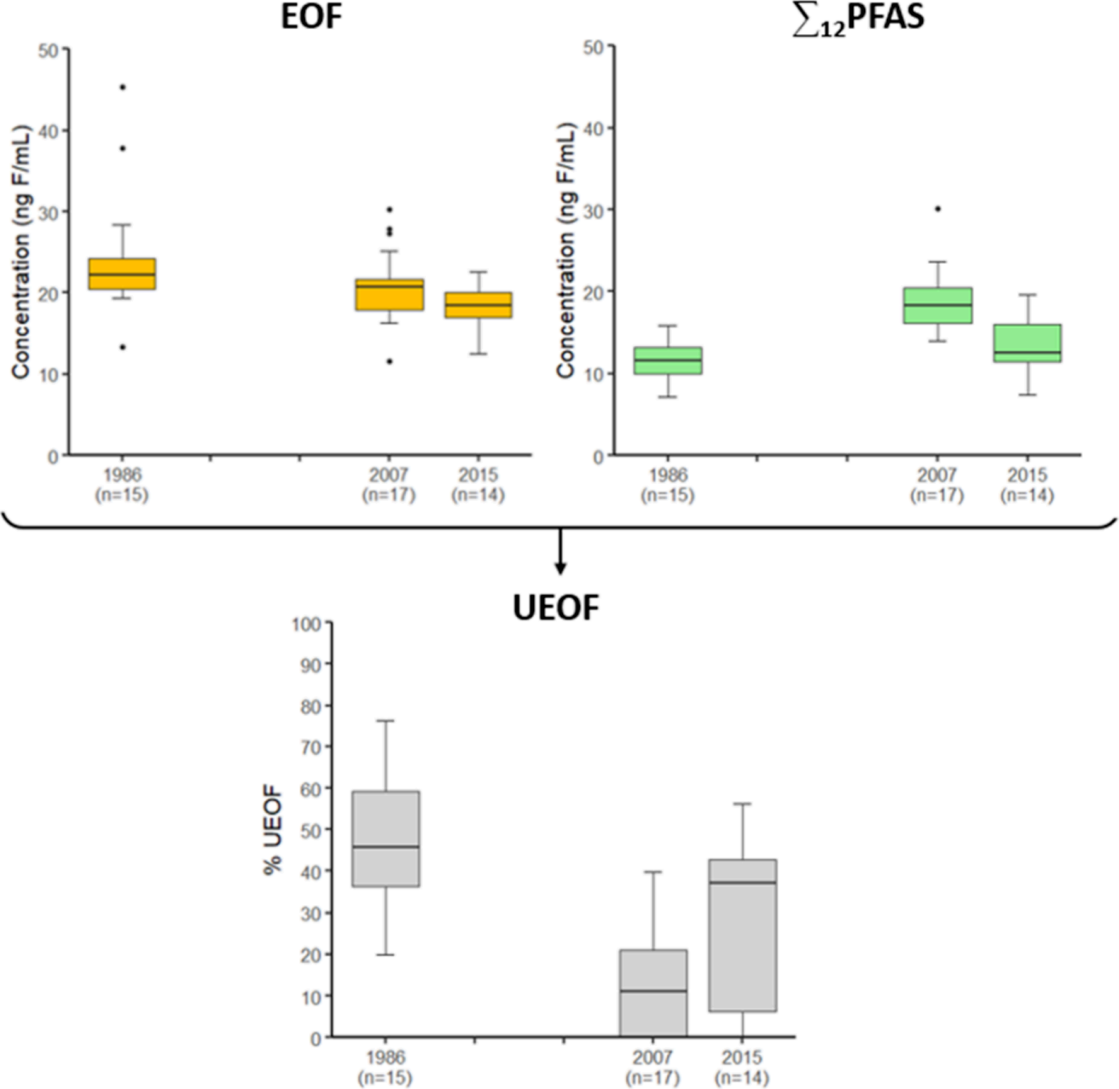
Comparison between extractable organic fluorine (EOF) and ∑_12_PFAS concentrations in ng F/mL and unidentified EOF (UEOF) percentage in pooled serum samples from the Tromsø Study in 1986, 2007, and 2015 (*n* = number of pools).

The comparison of EOF and target PFAS concentrations revealed the presence of unidentified organofluorine at all time-points. This unidentified EOF (UEOF) ranged from 0.00 to 34.8 ng F/mL, accounting for 0 to 77% of the EOF (Table S10, Figure 3).

UEOF concentrations were highest in 1986 when the target PFAS concentrations were lowest. In 2007, the UEOF portion was significantly lower than in 1986, while between 2007 and 2015, a significant increase in UEOF was observed (Figure 3, Table S8, Table S10).

For comparison, the UEOF fractions from other studies available in the literature are summarized in Table S10. While the UEOF in the Tromsø Study pools was higher in 1986 than in 2007, no time-trends were observed for the UEOF in German plasma between 1982 and 2000. The high fraction of UEOF observed in the 1986 Tromsø Study samples followed by lower concentrations in 2007 could be explained by the presence in the serum of PFOS-related substances that have been restricted with PFOS in the early 2000s. According to the PubChem PFAS Tree,^61^ there are 1297 chemicals registered in PubChem that would be restricted under Annex B of the Stockholm Convention. However, among these chemicals, C8-precursors can be excluded since no increases in PFOS and limited increases in PFOA were observed after the TOP assay in 1986 (Table 1). An increasing trend for UEOF following PFOS and PFOA production and use reduction has been observed between 2000 and 2009 plasma samples coming from Germany^14^ and in pooled serum samples from Swedish women, for which a 3.9% increase in UEOF per year between 1986 and 2017 has been modeled.^15^ The increase in UEOF that we observed between 2007 and 2015 (both in percentage and absolute concentration) is in agreement with these findings and could be explained by increasing exposure to novel PFAS that have not yet been identified.

However, fluorinated chemicals other than PFAS could also contribute to explain the elevated UEOF. Fluorine substitution is often used in the agrochemical and pharmaceutical industry. Among the halogenated agrochemicals available in the market between 1940 and 2003, around 28% contained fluorine.^62^ Meanwhile, for pharmaceuticals, the percentage of globally used active substances containing fluorine increased from around 2% in 1970 to 25% in 2021.^62,63^ This percentage is expected to increase further since 25–30% of the newly approved drugs contain one or more fluorine atoms. In addition, among the most prescribed drugs, the proportion of fluorinated pharmaceuticals is even higher.^62^ While we are not aware of studies investigating the contribution of pharmaceuticals and pesticides toward the EOF mass balance in human blood, a recent study determined that ∼22% of the EOF in wastewater treatment plant sludge (which mirrors societal use of chemicals) was attributable to these substances, many of which do not contain fluoroalkyl functionalities.^64^

**Figure 4:**
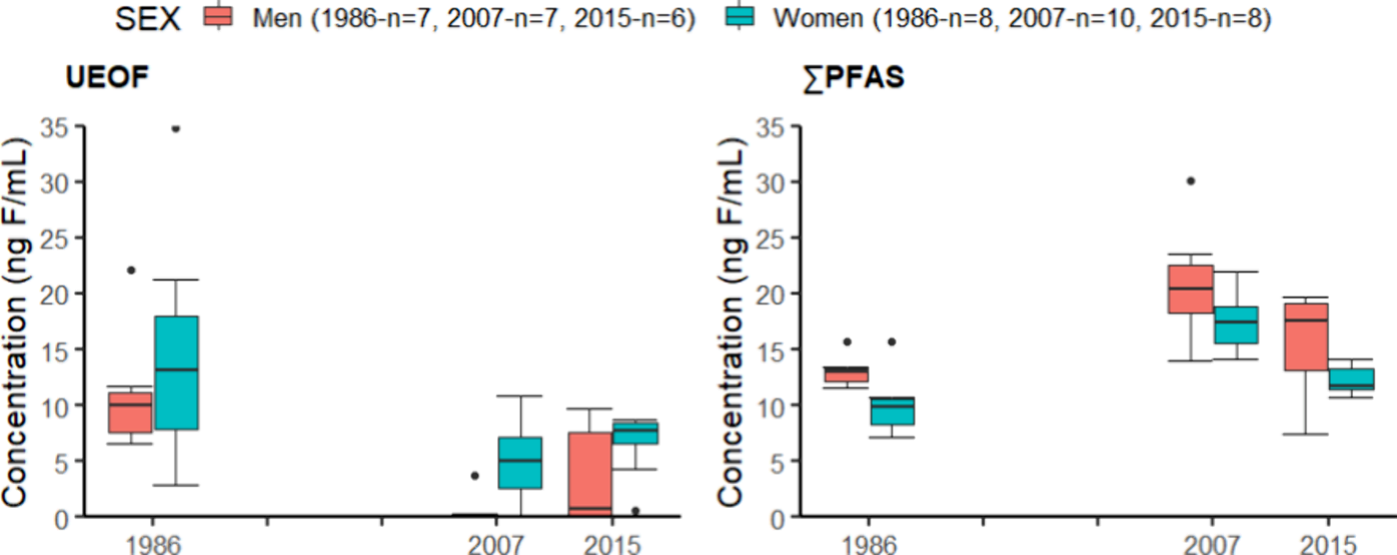
UEOF and ∑_12_PFAS in ng F/mL in men and women from the Tromsø Study in 1986, 2007, and 2015 (*n* = number of pools).

Mean age was not a significant predictor of UEOF, but women had higher UEOF values than men (Table S8, Figure 4). As for target PFAS, the evaluation of differences in concentrations between men and women at each time-point was limited by statistical power, and significant differences were observed only in 2007 (Table S9). The sex difference observed for UEOF is the opposite of what we observed and what is reported in the literature for PFAA, for which concentrations are higher in men than in women.^65−67^ Higher UEOF in women compared to men has also been reported in whole blood collected in Sweden, where the highest UEOF was reported in women aged 18–44.^16^ Two hypotheses were proposed by Aro et al.^16^ to explain the different UEOF concentrations between men and women. The first hypothesis is that a more frequent use of cosmetics and personal care products containing precursors (like PAPs) and other unknown PFAS^43,68^ could lead to higher blood concentrations of unknown PFAS or precursor metabolism intermediates that are not investigated in the target PFAS analyses. This hypothesis is also supported by studies reporting associations between PFAS concentrations in the blood and the use of cosmetics and personal care products.^69,70^ In our study, the TOP assay showed only a minor contribution of precursors to the EOF in human serum with no differences between men and women, and therefore, this first hypothesis regarding precursor exposure can be discarded. However, the more frequent use of cosmetics might still be a possible explanation for the higher UEOF in women compared to men since cosmetics could also lead to exposure to yet unknown PFAS that are not oxidizable and therefore nondetectable in the TOP assay. A second explanation could lie in a difference in use of fluorinated pharmaceuticals between men and women since sex differences in prescription are reported for several pharmaceuticals groups.^71−76^ Additionally, differences in elimination kinetics between men and women for these unidentified fluorinated chemicals could also play a role.

The TOP assay showed a limited contribution of oxidizable precursors to the EOF. The TOP ranged from 0.00 to 1.85 ng F/mL and accounted for a portion of the EOF ranging from 0 to 4% and for 0 to 100% of the UEOF. While the percentage contribution of TOP to the EOF remained the same in 1986 (median: 1%, range: 1–3%), 2007 (median: 1%, range: 0–4%), and 2015 (median: 1%, range: 0–2%), the contribution to the UEOF changed between time-points, ranging from 1 to 7% in 1986 (median: 2%), from 0 to 100% in 2007 (median: 18%), and from 0 to 37% in 2015 (median: 3%).

The TOP assay results suggest the absence of pharmaceuticals containing −CF_3_ groups since these should be oxidizable to TFA, which was not detected after oxidation. However, Hammel et al.^77^ found that among the 360 organofluorine pharmaceuticals approved and used globally between 1954 and 2021, 50% of these chemicals contained a single fluorine, 35% contained a single aromatic fluorine, and 10% contained more than three fluorine atoms. As most of these fluorinated pharmaceuticals contain only one fluorine, this large number of substances would go undetected in the TOP assay, and fluorinated pharmaceuticals could still contribute to the observed UEOF.

The EOF accounted for 20 to 99% of the TF and the unidentified TF (UTF) ranged from 5 to 1194 ng F/mL. This fraction did not change between time-points and was not influenced by sex and mean age (Table S8). The UTF can include both inorganic fluoride and organic fluorinated compounds not extracted or partially extracted with acetonitrile. Fasting plasma fluoride concentrations reported in the literature range from 9.3 to 24 ng F/mL in areas with nonfluorinated water (water fluoride concentrations <0.3 mg/L).^62,78^ Water in Norway is not fluorinated and a study from 2017 found that only 4 of 201 registered waterworks had fluoride exceeding the regulatory limit of 1.5 mg/L.^79^ In humans, the fluoride metabolism is not homeostatically regulated and plasma concentrations vary depending on levels of intake, deposition in hard tissues, and excretion.^80^ After ingestion, plasma concentrations take 3 to 6 h to return to baseline values.^78^ This could contribute to explain the variability observed in the UTF since the serum collected in the Tromsø Study is from nonfasting individuals. Overall, these observations indicate the need for measuring fluoride when conducting FMB studies using TF.

## 4 Implications and Limitations

The combined application of a set of targeted and group-wise analyses enabled the assessment of known and thus far unidentified organic fluorinated substances in human serum over three decades. No significant changes in TF were observed between 1986, 2007, and 2015. TF has the advantage of including both extractable and nonextractable fluorinated compounds. However, this advantage is lost if the fluoride contribution is not measured in human serum. Therefore, in this case, the EOF provides a better estimate of the overall exposure to organic fluorinated chemicals. The EOF concentrations were significantly higher in 1986 than those in 2007 and 2015. At the same time, the relative contributions of target PFAS and UEOF varied across the time-points examined. While target PFAS concentrations were highest in 2007, the highest UEOF concentrations were observed in 1986.

Interestingly, the UEOF concentrations were higher in women than in men, opposite of what is commonly observed for target PFAS. Differences in UEOF concentrations might reflect exposure to unknown PFAS, to fluorinated pharmaceuticals, and elimination kinetics for these yet unidentified chemicals. The difference in sex for UEOF deserves attention also because Kaiser et al.^18^ found UEOF in placental tissue and cord serum.

The addition of the TOP assay to FMB added valuable information about the contribution of PFAA precursors to human exposure. Precursors accounted only for 0–4% of the EOF, explaining a minor portion of the UEOF. However, it is important to highlight that the TOP assay provides only a lower bound estimate of precursor concentrations since conversion of precursors to PFAA can be incomplete.^32,81^ The TOP assay also provided key information on the structure of precursors, namely, minimal length of the perfluorinated carbon chain and presence of sulfur.

The UEOF found in pooled serum clearly indicates the need for additional tools to assess previously unidentified fluorinated compounds. The use of suspect and nontarget screening can be helpful in elucidating previously unidentified compounds. To close the FMB, these screening strategies should focus not only on PFAS but also on fluorinated pharmaceuticals and pesticides. In the present study, the lack of TFA increases after the TOP assay points to the absence of CF_3_-containing pharmaceuticals and pesticides. However, the yields of TFA from these chemicals in the TOP assay are not known yet, and many pharmaceuticals and pesticides containing a single fluorine cannot be detected with the TOP assay. Further studies are needed to understand the contribution of these chemicals to the EOF measured in human blood.

The use of pools instead of individual samples allowed for the screening of the Tromsø Study using the amounts of serum available from the biobank with a combination of multiple state-of-the-art analytical methods in a time- and cost-efficient manner. However, this was also a limitation since the effect of many variables known to influence PFAS exposure (e.g., dietary habits and parity) could not be assessed using pools. In addition, the individuals in each pool covered a wide range of ages, and this limited the investigation of the influence of age and birth cohorts on the different fluorine fractions measured.

## Supporting Information Available

Chemicals and consumables; characteristics of Tromsø Study samples and pools; quality control measures for TF, EOF, TOP assay, and target PFAS; data evaluation equations; PFAS concentrations used for FMB calculations; TF and EOF concentrations in human blood from this study and from the literature; multiple linear regression coefficients estimates and 95% confidence intervals for ln(TF), ln(EOF), ln(∑_12_PFAS), % UEOF, and TOP; multiple linear regression (including sex and sampling year interaction terms) coefficients estimates and 95% confidence intervals for ln(∑_12_PFAS) and % UEOF; UEOF concentrations in human blood from this study and from the literature; TF, EOF, TOP, ∑_12_PFAS, and UEOF concentrations in serum pools containing the same individuals in 1986, 2007, and 2015; UpSet plot showing the intersection of PFAA with increased concentrations after oxidation; individual target PFAS in pooled serum from 1986, 2007, and 2015; ∑_12_PFAS concentrations in relation with mean age of the individuals in the pools; individual target PFAS concentrations in men and women (PDF)
