# Supplementary material for: Fluorine Mass Balance, including Total Fluorine, Extractable Organic Fluorine, Oxidizable Precursors, and Target Per- and Polyfluoroalkyl Substances, in Pooled Human Serum from the Tromsø Population in 1986, 2007, and 2015
Source: Environ Sci Technol. 2023 Sep 25;57(40):14849–60. doi: 10.1021/acs.est.3c03655 (PMC10569050; doi:10.1021/acs.est.3c03655)
Supplement: Supplementary file 1 — es3c03655_si_001.pdf [file es3c03655_si_001.pdf]

Fluorine mass balance, including total fluorine, extractable organic fluorine, oxidizable precursors and target PFAS, in pooled human serum from the Tromsø population in 1986, 2007 and 2015

Lara Cioni[1,2], Merle Plassmann[3], Jonathan P. Benskin[3], Ana Carolina M. F. Coêlho[2], Therese H. Nøst[2], Charlotta Rylander[2], Vladimir Nikiforov[1], Torkjel M. Sandanger[1,2], Dorte Herzke[1,4*]

1. NILU, Fram Centre, Tromsø, Norway, NO-9296

2. UiT – The Arctic University of Norway, Department of Community Medicine, Tromsø, Norway, NO-9037

3. Stockholm University, Department of Environmental Science, Stockholm, Sweden, SE-106 91

4. Norwegian Institute for public Health, Oslo, Norway, NO-0213

***Corresponding authors**

Dorte Herzke - NILU, Fram Centre, Tromsø, Norway, NO-9296

*dhe@nilu.no

Summary: 21 pages, 6 figures 10 tables

The following information is included:

Chemicals and consumables (Page S3); characteristics of Tromsø Study samples and pools (Page S3-S4); quality control measures for TF, EOF, TOP assay and target PFAS (Page S4-S11); data evaluation equations (Page S11-S12); PFAS concentrations used for FMB calculations (Page S13); TF and EOF concentrations in human blood from this study and from the literature (Page S14); multiple linear regression coefficients estimates and 95% confidence intervals for ln(TF), ln(EOF), ln(∑12 PFAS), % UEOF and TOP (Page S14); multiple linear regression (including sex and sampling year interaction terms) coefficients estimates and 95% confidence intervals for ln(∑12 PFAS) and % UEOF (Page S15); UEOF concentrations in human blood from this study and from the literature (Page S15);TF, EOF, TOP, ∑12PFAS and UEOF concentrations in serum pools containing the same individuals in 1986, 2007 and 2015 (Page S16); UpSet plot showing the intersection of PFAA with increased concentrations after oxidation (Page S17); individual target PFAS in pooled serum from 1986, 2007 and 2015 (Page S18); $\sum_{12}$PFAS concentrations in relationship with mean age of the individuals in the pools (Page S19); individual target PFAS concentrations in men and women (Page S20).

1. Materials and methods

1.1. Chemicals and consumables

Acetonitrile (ACN, LiChrosolv®), tert-butyl methyl ether (MTBE, Suprasolv®), fuming hydrochloric acid (HCl, p.a. 37%) and sodium hydroxide (NaOH, EMSURE®, ≥ 99.0%) were obtained from Merck (Darmstadt, Germany). Sodium persulfate ($Na_2S_2O_8$, reagent grade, ≥ 98%, lot #BCCC8760) and ammonium acetate (NH_4OAc, LiChropur™) were obtained from Sigma-Aldrich (Steinheim, Germany). Ammonia (NH_3, solution 25%, AnalaR NORMAPUR) was purchased from VWR (Fontenay-sous-Bois, France). All native and isotopically labelled PFAS standards were obtained from Wellington Laboratories Inc. (Guelph, Ontario, Canada).

1.2. Serum samples and pooling strategy

Figure S1– Tromsø Study serum samples selection.

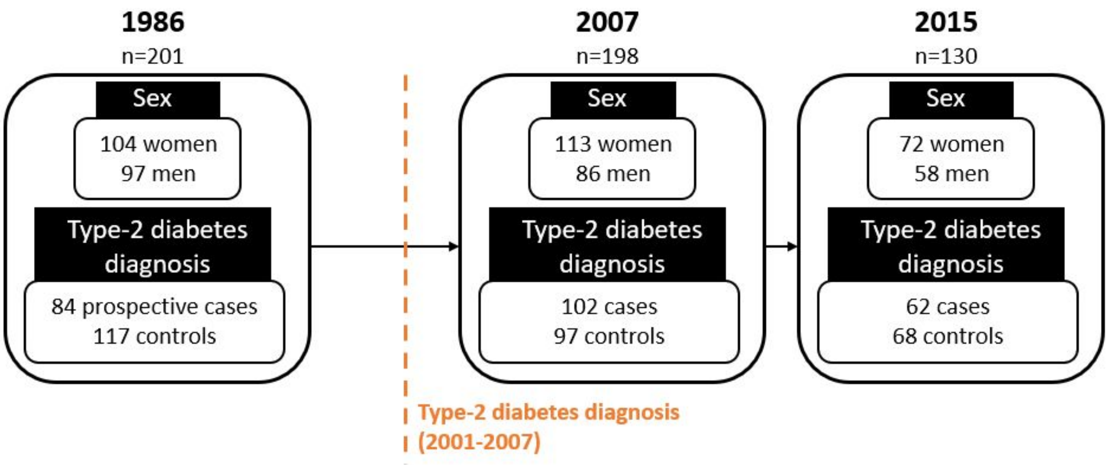

Table S1 – Characteristics of the Tromsø Study serum pools.

1986 (n=167)					2007 (n=175)					2015 (n=130)				
Pool ID	***n***	***Sex***	***Age mean (range)***	***Diabetes***	***Pool ID***	***n***	***Sex***	***Age mean (range)***	***Diabetes***	***Pool ID***	***n***	***Sex***	***Age mean (range)***	***Diabetes***
**1**	14	Women	36 (25-45)	Controls	**1**	14	Women	57 (46-66)	Controls	**1**	14	Women	65 (54-74)	Controls
**2**	12	Women	49 (46-57)	Controls	**2**	12	Women	70 (67-78)	Controls	**2**	12	Women	78 (75-86)	Controls
**3**	11	Women	41 (30-45)	Prospective cases	**3**	11	Women	62 (51-66)	Cases	**3**	11	Women	70 (59-74)	Cases
**4**	8	Women	49 (46-53)	Prospective cases	**4**	8	Women	70 (67-74)	Cases	**4**	8	Women	78 (75-82)	Cases
**5**	10	Men	34 (17-47)	Controls	**5**	10	Men	55 (38-68)	Controls	**5**	10	Men	63 (46-76)	Controls
**6**	10	Men	51 (48-55)	Controls	**6**	10	Men	72 (69-76)	Controls	**6**	10	Men	80 (77-84)	Controls
**7**	13	Men	44 (33-58)	Prospective cases	**7**	13	Men	65 (54-79)	Cases	**7**	13	Men	73 (62-87)	Cases
**8**	15	Women	31 (25-43)	Controls	**8**	15	Women	56 (46-64)	Controls	**8**	15	Women	63 (54-72)	Controls
**9**	15	Women	45 (43-47)	Controls	**9**	15	Women	67 (65-69)	Controls	**9**	15	Women	78 (74-82)	Controls
**10**	15	Women	52 (48-60)	Controls	**10**	15	Women	74 (70-81)	Controls	**10**	15	Women	70 (59-74)	Cases
**11**	15	Women	45 (43-48)	Prospective cases	**11**	15	Women	60 (51-64)	Cases	**11**	15	Women	78 (75-83)	Cases
**12**	15	Men	37 (17-49)	Controls	**12**	15	Women	67 (65-69)	Cases	**12**	15	Men	61 (46-73)	Controls
**13**	15	Men	55 (50-61)	Controls	**13**	15	Women	74 (70-81)	Cases	**13**	15	Men	79 (74-84)	Controls
**14**	15	Men	40 (25-48)	Prospective cases	**14**	15	Men	56 (38-66)	Controls	**14**	15	Men	81 (75-89)	Cases
**15**	15	Men	55 (49-60)	Prospective cases	**15**	15	Men	72 (68-76)	Controls					
					**16**	15	Men	62 (54-66)	Cases					
					**17**	15	Men	71 (67-76)	Cases					

Pool ID: cells highlighted in green indicate pools with same individuals across 1986, 2007 and 2015
n = number of individuals

1.3. TF quality control

A 9-point calibration curve ranging from 2.5 to 2500 ng of NaF in water (R^2>0.999) was included at the beginning and end of each run. Quality control measures for each run included: (1) three sample boat blanks for limit of detection (LOD) calculation, (2) two sample boats spiked with 100 ng of PFOS standard, and (3) three measurements of a certified reference material (fluorine in clay, CRM 461). Blanks ranged between 18 and 21 ng F/mL (n=9) and LOD (average boat blanks + 3 times the standard deviation of the blanks) ranged between 23 and 25 ng F/mL. The recovery of the PFOS standard (120 ± 6 %, n=6) confirmed complete

combustion and measurements of the certified reference material showed good accuracy and precision (recovery: 123 ± 9 %, n=9).

1.4. EOF quality control

For each extraction batch (14 serum samples), the quality control measures included: (1) three extraction blanks, (2) three reference serum samples not spiked, (3) one reference serum sample spiked with 239 ng of PFOS, (4) one reference serum sample spiked with 500 ng of NaF. The reference serum was obtained from the Arctic Monitoring and Assessment Programme (AMAP) Ring Test for Persistent Organic Pollutants [1]. Each extraction batch was run separately and included a calibration curve at the beginning and end of the run (2.5-1000 ng of NaF in water, R^2>0.999) and two sample boats spiked with 100 ng of PFOS standard. The extraction blanks ranged from 5 to 7 ng F/mL (n=12) and the EOF LOD (average extraction blanks + 3 times the standard deviation of the blanks) ranged from 6 to 9 ng F/mL. The analysis of the reference serum samples spiked with PFOS confirmed good recovery and reproducibility of the EOF analysis in human serum (recovery: 77 ± 14 %, n=8). The analysis of the controls spiked with NaF confirmed the removal of fluoride upon extraction (NaF recoveries ranging from 0 to 2 %, n=4).

1.5. TOP assay quality control

For each TOP assay batch (18 samples), a blank and an AMAP reference serum sample were included and processed as the samples. Blanks before and after oxidation showed low levels of PFAA (Table S2). LODs were calculated as the average concentration in the blanks plus 3 times the standard deviation of the blanks and in case of no detection in the blanks, LODs were calculated by multiplying the noise of the blanks by 3. LODs before and after oxidation were comparable for most compounds (Table S2). Measured PFAA concentrations before oxidation

in the AMAP serum samples were within -/+ 20% of the reference values. Mean recoveries before TOP assay ranged from 61 to 78 % and mean recoveries after TOP assay ranged from 55 to 65 %. Model precursors spiking oxidation experiments were performed as part of the validation described in our method paper and showed complete conversion for all spiked precursors and yields of PFAA ranging from 35-100% [2].

Table S2 - Average blank concentrations and LODs before and after TOP assay in ng/mL of serum (n=3).

Compound	Before TOP assay		After TOP assay	
	Blank concentration	LOD	Blank concentration	LOD
**PFBA**	0.15	0.47	0.12	0.49
**PFPeA**	0.20	0.32	0.26	0.47
**PFHxA**	0.03	0.10	0.13	0.39
**PFHpA**	0.01	0.02	0.02	0.13
**PFOA**	0.05	0.10	0.08	0.18
**PFNA**	0.00	0.02	0.01	0.03
**PFDA**	0.00	0.02	0.03	0.10
**PFUnDA**	0.00	0.02	0.00	0.04
**PFDoDA**	0.00	0.02	0.00	0.04
**PFTrDA**	0.00	0.07	0.00	0.09
**PFTeDA**	0.00	0.13	0.00	0.13
**PFBS**	0.00	0.04	0.02	0.08
**PFPeS**	0.00	0.07	0.00	0.07
**PFHxS**	0.10	0.13	0.01	0.04
**PFHpS**	0.00	0.03	0.00	0.03
**PFOS**	0.09	0.16	0.06	0.14
**PFNS**	0.00	0.04	0.00	0.04
**PFDS**	0.00	0.05	0.00	0.05

Table S3 – Recoveries in pooled serum samples before and after TOP assay (n=46).

Compound	Before TOP assay	After TOP assay
^{13}C-PFBA	74 ± 7	58 ± 10
^{13}C-PFPeA	78 ± 5	62 ± 5
^{13}C-PFHxA	75 ± 7	63 ± 4
^{13}C-PFHpA	70 ± 5	65 ± 4
^{13}C-PFOA	73 ± 6	62 ± 6
^{13}C-PFNA	71 ± 5	58 ± 5
^{13}C-PFDA	78 ± 5	55 ± 3
^{13}C-PFUnDA	61 ± 8	57 ± 5
^{13}C-PFDoDA	72 ± 6	61 ± 7
^{13}C-PFTeDA	75 ± 6	58 ± 7
^{13}C-PFHxS	74 ± 5	62 ± 6
^{13}C-PFOS	75 ± 3	61 ± 8

1.6. Target PFAS quality control

Target PFAS analyses on the EOF extracts included also the EOF extraction blanks (n=9). No PFAA were detected in the blanks and the LODs were calculated using the standard error of the regression divided the slope of the calibration curve multiplied by 3. LODs ranged from 0.03 to 0.13 ng/mL (Table S4). Measured PFAA concentrations in the AMAP serum samples use as quality control were within -/+ 20% of the reference values.

After the TOP assay the extracts were also analysed for C_2 and C_3-PFAA using a Raptor Polar X column. Trifluoroacetic acid (TFA) was analysed in a 5 minute isocratic run with 80 % 2mM ammonium acetate in methanol and 20 % 2mM ammonium acetate in 90:10 water:methanol. Perfluoropropionic acid (PFPrA), trifluoromethane sulfonic acid (TFMS), difluoro (perfluoromethoxy) acetic acid (1,2-PFECA), difluoroacetic acid (DiFA) and chlorodifluoro acetic acid (Cl-DiFA) were analysed in a 10 minute isocratic run using 80% 60:40

methanol:water with 0.05% formic acid and 20% 10 mM ammonium formate in water with 0.05% formic acid, based on an application note from Restek [3]. For these analyses, serum extracts were spiked with ^{13}C-TFA before oxidation and recoveries ranged from 56 to 65 % (n=46). Concentrations in the blanks ranged from 0.00 to 0.25 ng/mL. LODs were calculated as the average concentration in the blanks plus 3 times the standard deviation of the blanks and in case of no detection in the blanks, LODs were calculated by multiplying the noise of the blanks by 3 (Table S4).

133 **Table S4** – Target PFAS analysed on EOF extracts by UHPLC-Orbitrap.

Abbreviation	Name	LOD (ng/mL)
**PFCA (Perfluoroalkyl carboxylic acids)**		
PFBA	Perfluorobutanoic acid	0.07
PFPeA	Perfluoropentanoic acid	0.06
PFHxA	Perfluorohexanoic acid	0.07
PFHpA	Perfluoroheptanoic acid	0.07
PFOA	Perfluorooctanoic acid	0.06
PFNA	Perfluorononanoic acid	0.07
PFDA	Perfluorodecanoic acid	0.09
PFUnDA	Perfluoroundecanoic acid	0.10
PFDoDA	Perfluorododecanoic acid	0.10
PFTrDA	Perfluorotridecanoic acid	0.10
PFTeDA	Perfluorotetradecanoic acid	0.13
PFPeDA	Perfluoropentadecanoic acid	0.13
PFHxDA	Perfluorohexadecanoic acid	0.14
PFOcDA	Perfluorooctadecanoic acid	0.13
**PFSA (Perfluoroalkyl sulfonic acids)**		
PFBS	Perfluorobutane sulfonic acid	0.06
PFPeS	Perfluoropentane sulfonic acid	0.06
PFHxS	Perfluorohexane sulfonic acid	0.06
PFHpS	Perfluoroheptane sulfonic acid	0.06
PFOS	Perfluorooctane sulfonic acid	0.03
PFNS	Perfluorononane sulfonic acid	0.04
PFDS	Perfluorodecane sulfonic acid	0.05
PFUnDS	Perfluoroundecane sulfonic acid	0.06
**PFECA (Perfluoroalkyl ether sulfonic acids)**		
GenX	Ammonium perfluoro-4,8-dioxa-3H-nonanoic acid	0.08
ADONA	Perfluoro-4,8-dioxa-3H-nonanoic acid	0.08
**FTCA (Fluorotelomer carboxylic acids)**		
3:3 FTCA	3:3 Fluorotelomer carboxylic acid	0.06
5:3 FTCA	5:3 Fluorotelomer carboxylic acid	0.08
7:3 FTCA	7:3 Fluorotelomer carboxylic acid	0.08
**FTS (Fluorotelomer sulfonates)**		
4:2 FTS	4:2 Fluorotelomer sulfonic acid	0.06
6:2 FTS	6:2 Fluorotelomer sulfonic acid	0.08
8:2 FTS	8:2 Fluorotelomer sulfonic acid	0.08
**Perfluorooctane sulfonamido substances**		
FOSA	Perfluorooctane sulfonamide	0.07
Me-FOSA	N-Methyl perfluorooctane sulfonamide	0.07
Et-FOSA	N-Ethyl perfluorooctane sulfonamide	0.07
FOSAA	Perfluorooctane sulfonamidoacetic acid	0.06
Me-FOSAA	N-Methyl perfluorooctane sulfonamidoacetic acid	0.06
Et-FOSAA	N-Ethyl perfluorooctane sulfonamidoacetic acid	0.06
Me-FOSE	N-Methyl perfluorooctane sulfonamido ethanol	0.08
Et-FOSE	N-Ethyl perfluorooctane sulfonamido ethanol	0.08
**Cl-PFAES**		
9Cl-PF3ONS	9Cl-Perfluoro-3-oxononane sulfonic acid	0.10
11Cl-PF3OUdS	11Cl-Perfluoro-3-oxoundecane sulfonic acid	0.10
**PAPs**		
4:2 monoPAP	4:2 Fluorotelomer phosphate monoester	0.10
4:2 diPAP	4:2 Fluorotelomer phosphate diester	0.10
6:2 monoPAP	6:2 Fluorotelomer phosphate monoester	0.10
6:2 diPAP	6:2 Fluorotelomer phosphate diester	0.10
6:2/8:2 diPAP	6:2/8:2 Fluorotelomer phosphate diester	0.10
6:2/10:2 diPAP	6:2/10:2 Fluorotelomer phosphate diester	0.12
6:2/12:2 diPAP	6:2/12:2 Fluorotelomer phosphate diester	0.12

6:2/14:2 diPAP	6:2/14:2 Fluorotelomer phosphate diester	0.12
8:2 diPAP	8:2 Fluorotelomer phosphate diester	0.13
8:2/10:2 diPAP	8:2/10:2 Fluorotelomer phosphate diester	0.13
10:2 monoPAP	10:2 Fluorotelomer phosphate monoester	0.13
10:2 diPAP	10:2 Fluorotelomer phosphate diester	0.13

Table S5 - Average blank concentrations and LODs before and after TOP assay in ng/mL of serum (n=3).

Compound	Blank concentration	LOD
**TFA**	0.28	0.32
**PFPrA**	0.10	0.13
**TFMS**	0.00	0.07
**1,2-PFECA**	0.00	0.07
**DiFA**	0.00	0.07
**Cl-DiFA**	0.00	0.07

1.7. Data evaluation

For comparison with EOF values, target PFAS concentrations measured in the EOF extracts and ΔPFAA concentrations from the TOP assay were converted to F equivalents using the following equation:

$$Concentration\left(\frac{ng\ F}{mL}\right) = \frac{concentration\left(\frac{ng}{mL}\right)\cdot \mathrm{nF} \cdot AW_F}{MW_{PFAS}} \quad \text{(S1)}$$

where nF is the number of fluorine atoms in the PFAS structure, A_F is the atomic weight of fluorine and MW_{PFAS} is the molecular weight of the PFAS which concentration is being converted.

Differences in TF, EOF, $\sum_{12}$ PFAS, unidentified EOF and TOP between sampling years were assessed by multiple linear regression to account for the influence of sex and age (as weighted mean of the age of the individuals in the pools expressed in years) using the following equation:

$$y = \beta_0 + \beta_1 dummy\,1 + \beta_2 dummy\,2 + \beta_3\,sex + \beta_4\,age \qquad \text{(S2)}$$

where y is the log transformed concentration for TF, EOF and $\sum_{12}$ PFAS, the ΔPFAA concentration in ng/mL for TOP and the percentage contribution to EOF for UEOF; β0 is the intercept of the multiple linear regression; β_1, β_2, β_3 and β_4 are the regression coefficients for the predictor variables; dummy 1 is a dummy variable equal to 1 if sampling year is 1986, equal to 0 if sampling year is 2007 or 2015; dummy 2 is a dummy variable equal to 1 if sampling year is 2015, equal to 0 if sampling year is 1986 and 2007; sex is categorical variable equal to 0 for women and equal to 1 for men; age is the weighted mean age of the individuals making up each pool expressed in years.

When sex was a significant predictor, differences in concentrations between men and women at each sampling year were assessed by adding an interaction term between sex and each sampling year dummy variable as described by equation S3.

$$y = \beta_0 + \beta_1 dummy\,1 + \beta_2 dummy\,2 + \beta_3\,sex + \beta_4\,age + \beta_5\,dummy1\,sex + \beta_6\,dummy2\,sex \qquad \text{(S3)}$$

2. Results

Table S6 – PFAS concentrations (ng/mL) used for fluorine mass-balance calculations (concentrations are not recovery corrected).

	1986 (n=15)				2007 (n=17)				2015 (n=14)			
	DF	Median	Mean	Range	DF	Median	Mean	Range	DF	Median	Mean	Range
**PFHpA**	0/15	-	-	-	0/17	-	-	-	0/14	-	-	-
**PFOA**	15/15	1.60	1.50	0.88-2.04	17/17	2.32	2.40	2.00-2.96	14/14	1.52	1.56	1.12-2.24
**PFNA**	15/15	0.24	0.25	0.08-0.64	17/17	1.04	0.99	0.72-1.52	14/14	1.14	1.18	0.68-1.60
**PFDA**	1/15	<0.09	<0.09	<0.09-0.24	17/17	0.40	0.39	0.20-0.84	14/14	0.50	0.50	0.20-0.84
**PFUnDA**	15/15	0.32	0.32	0.12-0.56	17/17	0.60	0.60	0.24-1.56	14/14	0.62	0.58	0.24-1.20
**PFDoDA**	0/15	-	-	-	0/17	-	-	-	0/14	-	-	-
**PFHxS**	15/15	0.40	0.38	0.16-0.72	17/17	1.44	1.59	1.04-4.68	14/14	1.24	1.36	0.72-3.12
**PFHpS**	4/15	<0.03	<0.03	<0.03-0.08	17/17	0.20	0.17	0.04-0.36	14/14	0.08	0.09	0.04-0.20
**br-PFOS**	15/15	3.68	3.58	2.48-5.16	17/17	5.44	5.26	3.68-7.16	14/14	3.14	3.40	2.56-4.88
**lin-PFOS**	15/15	10.9	10.4	6.72-15.4	17/17	16.2	16.5	11.1-30.2	14/14	10.4	11.3	5.52-18.8
**FOSAA**	9/15	0.08	0.08	<0.06-0.20	0/17	-	-	-	0/14	-	-	-
**Me-FOSAA**	14/15	0.16	0.13	<0.06-0.28	10/17	0.08	0.08	<0.06-0.20	0/14	-	-	-
**Et-FOSAA**	15/15	0.28	0.27	0.12-0.52	0/17	-	-	-	0/14	-	-	-
**∑ 12 PFAS**	15/15	17.8	17.2	11.0-24.1	17/17	27.9	28.3	21.5-46.1	14/14	19.2	20.3	11.4-30.0

DF = detection frequency: number of pools with PFAS concentration > LOD.

Table S7 – Descriptive statistics for TF and EOF concentrations (ng F/mL) in the Tromsø Study pooled serum samples from 1986, 2007 and 2015 and in samples from previous studies available in the literature (n=number of pools/number of individual samples).

Study	Country	Sampling year	Matrix	n	TF (ng F/mL)			EOF (ng F/mL)		
					Median	Mean	Range	Median	Mean	Range
This study	Norway	1986	Serum (pooled)	15	79.1	112	<25.0-1330	22.2	23.3	13.3-45.3
This study	Norway	2007	Serum (pooled)	17	74.2	74.8	<25.0-1212	20.8	20.5	16.2-30.3
This study	Norway	2015	Serum (pooled)	14	68.3	71.6	<25.0-265	18.5	18.4	12.6-22.6
Miyake et al. (2007)	Japan	2003-2004	Whole blood	3	208	214	181-262	<6	<6	<6-8.89
Miyake et al. (2007)	USA	2001	Plasma	4	149	163	140-189	45.2	38.3	17.8-59.0
Yeung et al. (2008)	China	2004	Whole blood	30	-	-	60.6-166	-	-	<6-43.4
Yeung and Mabury (2016)	China	2004	Whole blood	34	-	-	-	17	18.4	8.22-94.4
Yeung and Mabury (2016)	Germany (Halle)	1995-2009	Plasma	42	-	-	-	-	15.9	5.29-43.9
Yeung et al. (2016)	Germany (Munster)	1982-2009	Plasma	80	-	-	-	-	23.7	9.20-115
Miaz et al. (2020)	Sweden	1996-2017	Serum (pooled)	57	-	-	-	-	-	8.10-32.0
Aro et al. (2021)	Sweden	2015	Whole blood	9	-	-	-	-	24.8	17.6-37.8
Aro et al. (2021)	Sweden (Ronneby)	2014-2016	Whole blood	20	-	-	-	-	234	<107-592
Aro et al. (2021)	Sweden	2018-2019	Whole blood	130	-	-	-	-	-	0.51-48.7
Kaiser et al. (2021)	Austria	2021	Serum (pooled)	6	-	-	-	-	3.83	2.85-7.17

Table S8 – Multiple linear regression coefficients estimates and 95% confidence intervals for ln(TF), ln(EOF), ln(∑12 PFAS), % UEOF and TOP in pooled serum samples from the Tromsø Study.

	ln(TF)	ln(EOF)	ln((∑12 PFAS)	% UEOF	TOP
	Estimate (95% CI)	Estimate (95% CI)	Estimate (95% CI)	Estimate (95% CI)	Estimate (95% CI)
β_0 (intercept)	1.17 (2.68 to 5.03)	2.55 (1.91 to 3.20)	2.76 (2.33 to 3.08)	61.4 (18.8 to 104)	0.49 (-0.48 to 1.45)
β_1 (1986-2007)	1.41 (-0.15 to 2.97)	0.29* (0.03 to 0.55)	-0.48*** (-0.61 to -0.35)	22.8* (5.60 to 40.0)	0.14 (-0.25 to 0.53)
β_2 (2015-2007)	-0.39 (-1.47 to 0.68)	-0.16 (-0.34 to 0.02)	-0.41*** (-0.51 to -0.30)	18.2** (6.35 to 30.0)	0.02 (-0.24 to 0.29)
β_3 (sex)	0.04 (-0.77 to 0.85)	-0.05 (-0.18 to 0.09)	0.18*** (0.10 to 0.26)	-14.3** (-23.3 to -5.32)	-0.16 (-0.36 to 0.05)
β_4 (age mean)	0.05 (-0.01 to 0.11)	0.01 (-0.002 to 0.017)	0.02*** (0.01 to 0.02)	-0.64 (-1.27 to 0.01)	-0.001 (-0.015 to 0.013)
R^2	0.084	0.209	0.796	0.594	0.091
F-test p-value	0.445	0.042	0.000	0.000	0.409

*p < 0.05
**p < 0.01
*** p < 0.001

Table S9 – Multiple linear regression (including sex and sampling year interaction terms) coefficients estimates and 95% confidence intervals for ln(∑12 PFAS) and % UEOF in pooled serum samples from the Tromsø Study.

	**ln((∑12 PFAS)**	**% UEOF**
	Estimate (95% CI)	Estimate (95% CI)
β_0 (intercept)	2.72 (2.34 to 3.11)	64.4 (20.3 to 109)
β_1 (1986-2007)	-0.17 (-0.35 to 0.01)	18.3 (-2.27 to 38.8)
β_2 (2015-2007)	-0.40*** (-0.53 to -0.27)	17.1* (1.91 to 32.3)
β_3 (2007 sex)	0.16* (0.02 to 0.28)	-18.3* (-33.4 to -3.14)
β_4 (age mean)	0.02*** (0.01 to 0.02)	-0.66* (-1.31 to -0.01)
β_5 (1986 sex)	0.08 (-0.11 to 0.27)	9.24 (-12.7 to 31.2)
β_6 (2015 sex)	-0.01 (-0.02 to 0.18)	3.04 (-19.4 to 25.5)
R^2	0.802	0.602
F-test p-value	0.000	0.000
*p < 0.05<br>**p < 0.01<br>*** p < 0.001		

Table S10 – Descriptive statistics for UEOF concentrations (ng F/mL and/or %) in the Tromsø Study pooled serum samples from 1986, 2007 and 2015 and in samples from previous studies available in the literature (n=number of pools/number of individual samples).

**Study**	**Country**	**Sampling year**	**Matrix**	**n**	**UEOF**		
					Median	Mean	Range
This study	Norway	1986	Serum (pooled)	15	10.5 ng F/mL<br>46%	10.9 ng F/mL<br>46%	2.93-34.8 ng F/mL<br>21-77%
This study	Norway	2007	Serum (pooled)	17	2.26 ng F/mL<br>10%	3.17 ng F/mL<br>14%	0.00-10.9 ng F/mL<br>0-40%
This study	Norway	2015	Serum (pooled)	14	7.54 ng F/mL<br>37%	5.32 ng F/mL<br>27%	0.00-9.74 ng F/mL<br>0-56%
Miyake et al. (2007)	Japan	2003-2004	Whole blood	3	-	-	0.00-1.38 ng F/mL<br>0-15%
Miyake et al. (2007)	USA	2001	Plasma	4	-	-	0.00-4.40 ng F/mL<br>0-15%
Yeung et al. (2008)	China	2004	Whole blood	30	-	-	15-43%
Yeung and Mabury (2016)	China	2004	Whole blood	34	-	-	14-69%
Yeung and Mabury (2016)	Germany (Halle)	1995-2009	Plasma	42	-	-	0.0-9.5 ng F/mL
Yeung et al. (2016)	Germany (Munster)	1982-2009	Plasma	80	-	-	0.0-9.9 ng F/mL
Miaz et al. (2020)	Sweden	1996-2017	Serum (pooled)	57	-	-	11-75%
Aro et al. (2021)	Sweden	2015	Whole blood	9	-	84%	71-97%
Aro et al. (2021)	Sweden (Ronneby)	2014-2016	Whole blood	20	-	37%	0-76%
Aro et al. (2021)	Sweden	2018-2019	Whole blood	130	-	0-99%	-
Kaiser et al. (2021)	Austria	2021	Serum (pooled)	6	-	1.17 ng F/mL<br>24%	-

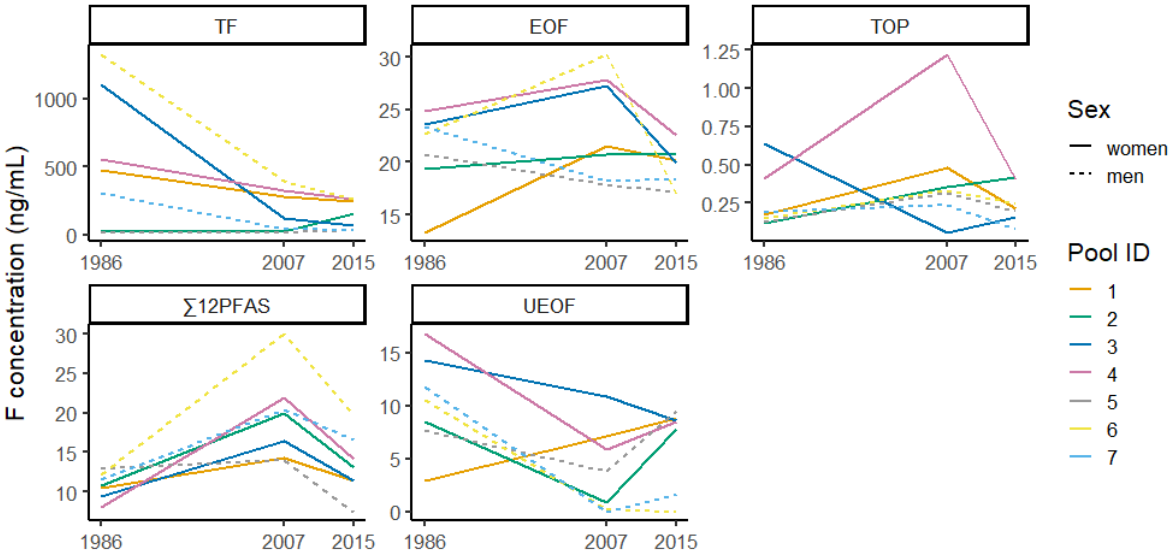

195

196 **Figure S2** – TF, EOF, TOP, $\sum_{12}$PFAS and UEOF concentrations (ng F/mL) in serum pools
197 from the Tromsø Study containing the same individuals in 1986, 2007 and 2015.

198

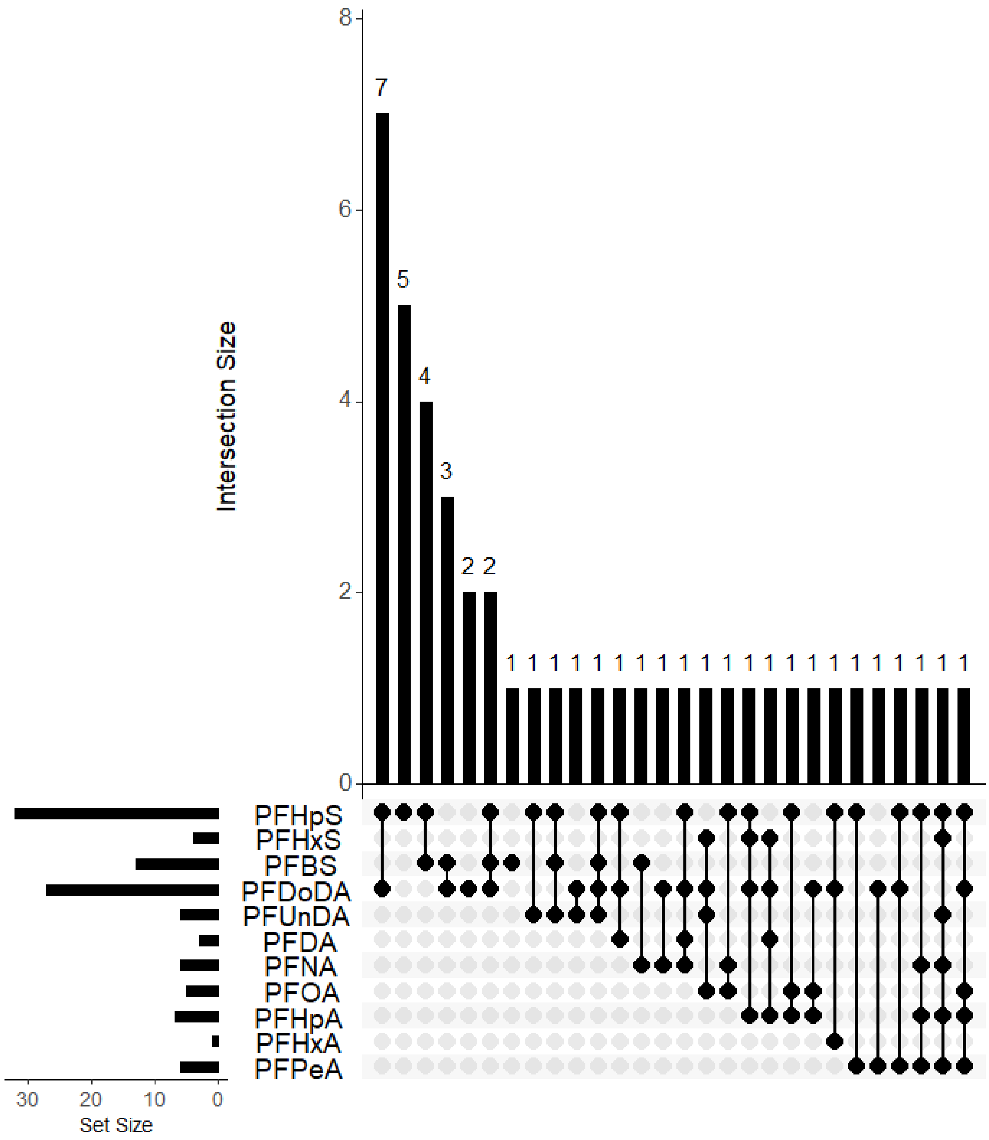

Figure S3 - UpSet plot showing the intersection of PFAA with increased concentrations after oxidation. The bar chart shows the number of pools with increases in concentrations of a combination of PFAA. The graphical table underneath indicates the PFAA combinations (black dots and lines). The frequency count of each PFAA across all subsets is shown as a smaller bar chart on the left side of the graphical table.

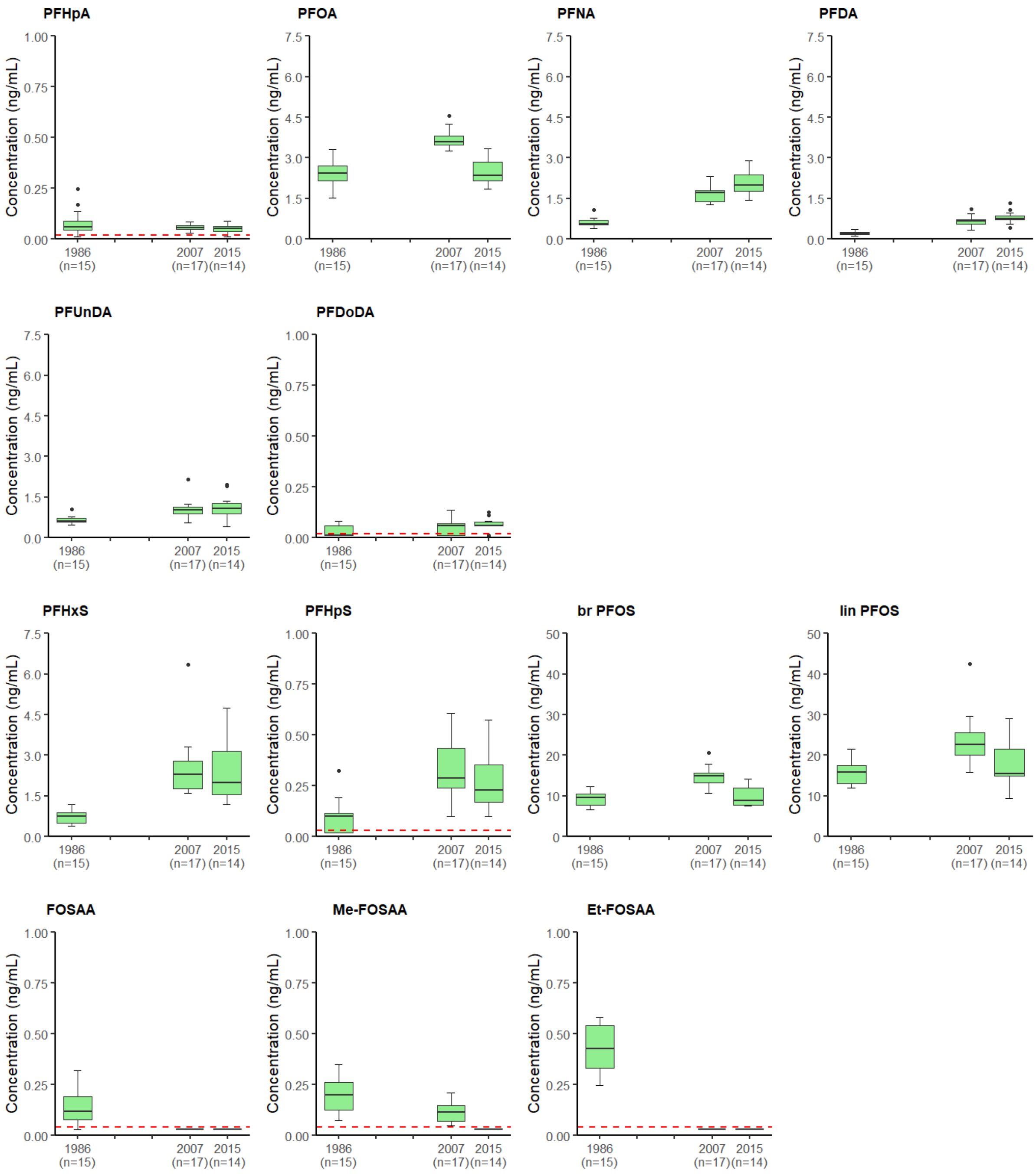

Figure S4 – Target PFAS (ng/ml) in pooled serum samples from the Tromsø Study collected in 1986, 2007 and 2015.

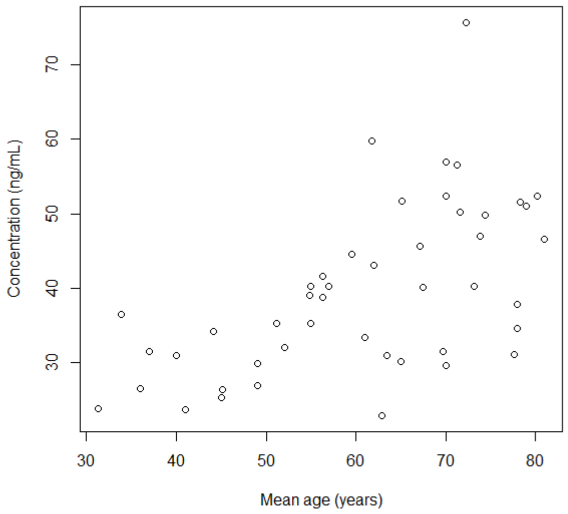

Figure S5 – Concentrations of ∑12 PFAS in pooled serum samples from the Tromsø Study collected in 1986, 2007 and 2015 in relationship with mean age of the individuals in the pools in years.

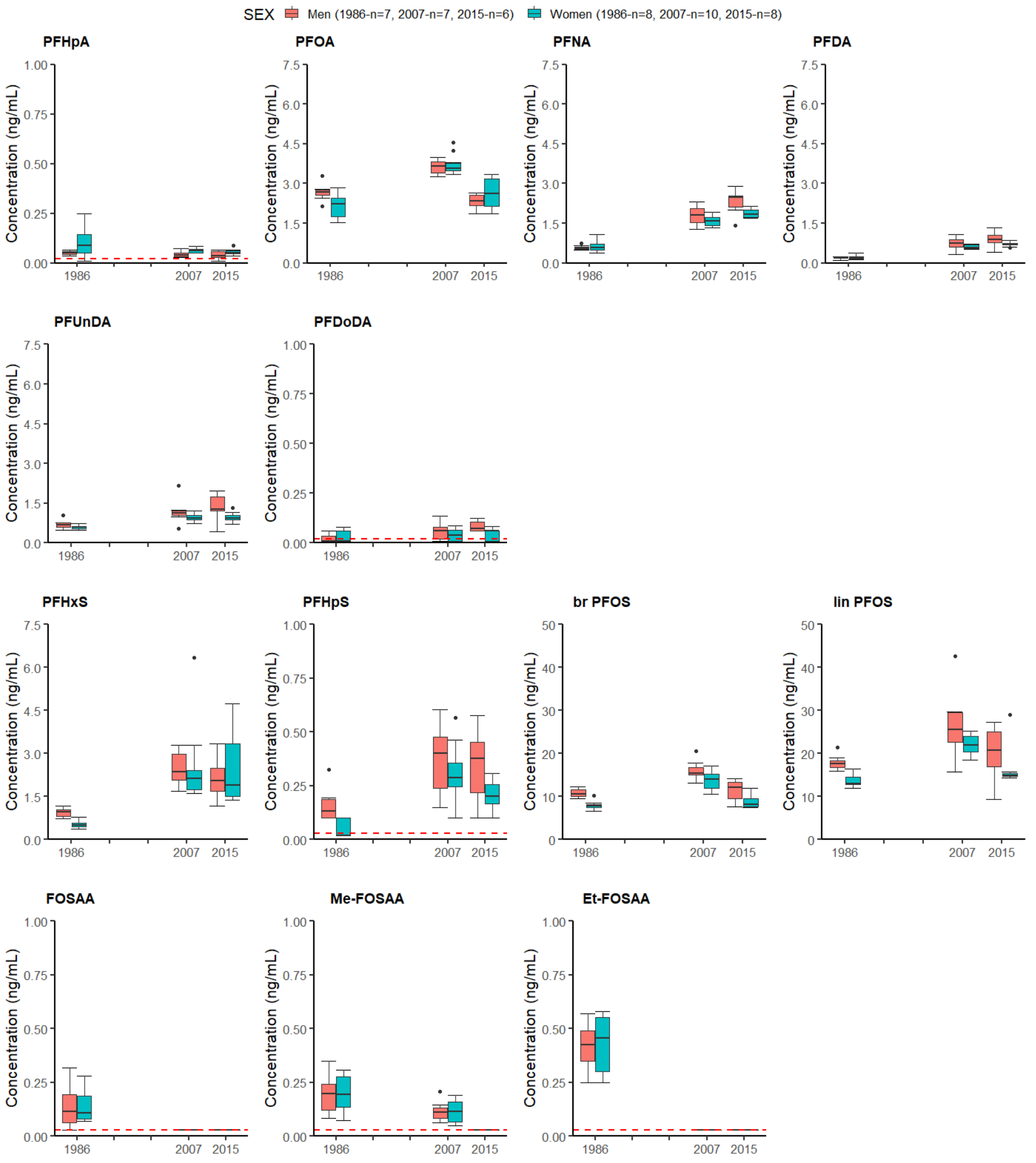

Figure S6 – Target PFAS concentrations (ng/mL) in men and women from the Tromsø Study in 1986, 2007 and 2015.

References

1. AMAP, *AMAP Assessment 2021: Human Health in the Arctic.* 2022, Arctic Monitoring and Assessment Programme (AMAP): Tromsø, Norway. p. 240.
2. Cioni, L., et al., *Total Oxidizable Precursors Assay for PFAS in Human Serum.* Environment International, 2022(170).
3. Restek. *Raptor Polar X: Separate a Wide Variety of Polar Analytes with a Novel Hybrid Stationary Phase* https://www.restek.com/row/technical-literature-library/articles/raptor-polar-x-separate-a-wide-variety-of-polar-analytes-with-a-novel-hybrid-stationary-phase/. 2020.
